# Association of healthy eating index (2015) with depression and anxiety symptoms among Iranian adolescent girls

**DOI:** 10.1186/s41043-024-00529-z

**Published:** 2024-04-02

**Authors:** Elham Ghanbarzadeh, Ahmad Reza Dorosty Motlagh, Behnood Abbasi

**Affiliations:** 1 Department of Nutrition, Electronic Health and Statistics Surveillance Research Center, Science and Research Branch, Islamic Azad University, Tehran, Iran; 2 Department of Community Nutrition, School of Nutritional Sciences and Dietetics, Tehran University of Medical Sciences, Tehran, Iran

**Keywords:** Healthy eating index, Depression, Anxiety, Adolescents

## Abstract

Adolescence is a period of rapid growth, with changes in body composition and cognitive and psychosocial development. Teenagers who eat properly and participate in daily physical activities have a healthy lifestyle. Healthy living promotes optimal growth and performance at school and in the workplace and minimizes the risk of chronic nutrient-related diseases. Therefore, the present study was conducted to determine the relationship between the healthy eating index (2015) (HEI-2015) and depression and anxiety among Iranian adolescent girls. This cross-sectional study was designed based on the updated version of HEI-2015. The study population consisted of 412 high school girls aged 12–17 years old. Data were collected about the diet, sociodemographic, and anthropometric characteristics of the participants. HEI and anthropometric characteristics of the participants were measured. The depression, anxiety, and stress scale 42 (DASS-42) questionnaire was used to detect adolescents suffering from depression and anxiety. The relationships of the HEI and anthropometric measures with depression and anxiety were also assessed. The results showed that the HEI is inversely correlated with depression and anxiety in Iranian adolescent girls. HEI was greater in the healthy participants than in those suffering from depression and anxiety (*P* < 0.0001).

## Novelty

This is the first investigation into the relationship between HEI and depression and anxiety in adolescents.

The results depicted an inverse relationship between HEI and depression.

The results showed that the HEI is inversely correlated with anxiety.

HEI was greater in the healthy participants than in those suffering from depression and anxiety.

## Introduction

Adolescence is a sensitive period during which one experiences physical, social, and mental changes caused by the increase in the level of pubertal hormones [1]. According to statistics, approximately 20% of children and adolescents worldwide suffer from mental health problems, including depression, annually. Depression is ranked as the second most common cause of death in adolescents by suicide [2]. On the other hand, according to the statistics of the Institute of Health Metrics and Evaluation, in 2021, 58 million children and adolescents were living with anxiety disorders [3]. Anxiety disorders are known as excessive fear and worry and associated behavioral disorders. Sometimes the symptoms are so severe that significant impairment or dysfunction is observed in the person [4]. Because mental health problems often begin in childhood or adolescence, they are associated with other developmental and health conditions that affect quality of life, personality disorders, social and academic function, and substance abuse [5], as well as weight problems in adulthood [6]. In Iran, according to the Epidemiological Research Center’s Depression Scale (CES-D), the major depression rate for 670 female students aged 15–18 in 2019 was 52.6 [7]. According to another study, the prevalence of anxiety in girls (21.8%) was higher than that in boys (11.6%), and the prevalence of depression in boys (29.5%) was higher than that in girls (17.8%) [8].

A large body of evidence has shown associations between dietary patterns, food, and nutrient intake and mental health, including depression and anxiety [9–12]. A bidirectional relationship has been observed between dietary intake and symptoms of depression and anxiety. Based on one study, people with depression and anxiety often have poor eating habits [13]. According to studies, dietary patterns, including traditional and Mediterranean diets, fruit and vegetable consumption, and intake of some nutrients, including omega-3 fatty acids [14], folate, vitamin D [15], B12 [16], Zn, Se, and Fe [17–19] have been associated with a reduced incidence of depression [20–22] and some dietary groups, including vegetables, fruits, and fish, improve depression problems [23]. Additionally, diets rich in fruits, vegetables, and whole grains are inversely related to anxiety, while those rich in sweets, beverages, red meat, and processed foods increase the chances of developing anxiety [24, 25]. Food and diet patterns affect the structure, neurotransmitters, and functions of the brain, which subsequently impact human behaviors [26–28]. Studies have shown that diet affects reward-seeking behaviors, behavioral restraints, learning changes, memory, and adolescent cognition [29], but these changes were not observed during adulthood [30, 31]. At the same time, it has been observed that the consumption of processed foods and fat and sugar-rich diets has increased in the last 50 years, which is associated with an increase in obesity, diabetes, and cognitive and emotional disorders [32, 33]. Accordingly, poor-quality diets and malnutrition are more commonly associated with mental health problems [34, 35]. An Australian study showed that adolescents on a healthy diet are less likely to report symptomatic depression and anxiety, but those with processed “junk food” are more likely to report depression [1]. In addition, adolescents with a history of malnutrition are more likely to experience behavioral problems and depression during the critical phase of childhood brain development [36]. Another approach is concerned with the effect of overall diet and eating habits on mood. According to this approach, a Western or unhealthy diet is associated with mental health disorders [37] whereas a “healthy” or “good-quality” diet is related to better mental health [38, 39]. The Healthy Eating Index-2015 (HEI-2015), the newer version of the HEI-2010, is a valid and reliable measure of dietary quality that complies with the 2015–2020 dietary guidelines [40]. In a study conducted on 1118 participants in the United States. An inverse relationship was observed between the HEI-2010 and depression [41]. Another study in Iran showed that subjects who adhered to the Alternative Healthy Eating Index (AHEI-2010) had a reduced likelihood of anxiety and depression to 49% and 45%, respectively [42].

In Iran, few studies have reported the relationship between adolescent diet and symptoms of depression or anxiety. Therefore, considering that the issue of nutrition and mental health is a public health concern and that there is little evidence in this field, the present study investigated the relationship between dietary intake and symptoms of depression and anxiety in adolescent girls in Iran.

## Methodology and participants

### Participants and study design

In this cross-sectional-analytical study, 420 schoolgirls aged 12 to 17 were randomly selected from 52 public high schools in Babol, Mazandaran, Iran, from February 2019 to May 2020. The sample size was calculated using the formula

$$\text{n}=\frac{{(\text{z}}_{1-\raisebox{1ex}{${\upalpha }$}\!\left/ \!\raisebox{-1ex}{$2$}\right.}+{\text{z}}_{1-{\upbeta }})2 \overline{\text{p}\text{q}}}{{(\text{p}}_{1}-{\text{p}}_{2})2}$$

as it is in

$$\overline{\text{p}}=\frac{{(\text{p}}_{1}+{\text{p}}_{2}) }{2} \;\text{a}\text{n}\text{d}\; \overline{\text{q}}=1- \overline{\text{p}}$$

considering α = 0.05 and β = 0.2, the results of P1 and P2 from the pilot study are obtained as follows: In the pilot study that was conducted on 30 people and to estimate P1 and P2, the value of P1 (probability of inappropriateness of HEI in depressed people) was 0.53, and P2 (probability of inappropriateness of HEI in healthy people) was found to be 0.47. (This method was also calculated separately for anxious people. Since the number of samples in depressed and healthy people with inappropriate HEI was more than in the other cases, it was used to determine the sample size.) By replacing the above items in the formula, the sample number was 403 people. Considering the possibility of people leaving the study, 420 students were examined in this study. Since 8 students did not complete the questionnaires completely, they were excluded from the study, and 412 students were examined. Having no diet or specific allergies, lack of physical illness, and lack of use of certain medications were the inclusion criteria. Participants’ socioeconomic status (SES) was assessed using a demographic questionnaire. All participants in this study were given informed consent to participate in the study and were approved for research ethics (IR.IAU.SRB.REC.1400.229).

### Assessment of dietary intake and HEI-2015

A 24-hour food recall questionnaire was used for nutrient intake assessment by converting food intake into micronutrients and total calorie intake. The 24-hour recall questionnaire was concocted for three consecutive days including two weekdays and one weekend. They were completed through face-to-face interviews with participants for weekday questionnaires, and self-report was then returned to the researcher by participants for weekends questionnaires. Then, all the eaten food was converted into consumed heat (calories) using the home scale table. We then used Nutritionist IV (NSquared Computing, Tehran), a software modified for the Iranian population, to calculate the amount (in grams) of all nutrients and food groups. HEI-2015 compliance was determined using recommended guidance [43]. With this method, points are awarded based on 13 food groups. The nine validity components of HEI-2015 include total fruits, whole fruits, total vegetables, greens and beans, whole grains, dairy, total protein foods, seafood, plant protein, and fatty acids (polyunsaturated fatty acid + monounsaturated fatty acids)/(saturated fatty acid). It also consists of four moderated components, including refined grains, sodium, added sugars, and saturated fats. Similar to HEI-2005 and HEI-2010, each of these components is evaluated based on a density of 1,000 calories except fatty acids, which is the ratio of unsaturated to saturated fatty acids. For adequacy components, the minimum and maximum intakes can range from 0 to 5, respectively. However, for dairy products, whole grain products, and fatty acids, the maximum achievable score is 10 considering the moderation component, and the input range is 0–10 (the lowest intake gets 10 points and the maximum intake gets 0 points). To calculate the HEI-2015 sum, the scores were obtained from each participant for 13 foods, and the participants were placed in quantiles based on the results.

### Depression and anxiety evaluation

Depression and anxiety conditions were assessed using the Depression, Anxiety, and Stress Scales-42 questionnaire (DASS-42) which consists of three categories of 7-item self-report scales [44]. This tool has already been approved for the Iranian population [45]. In the current study, the anxiety and depression sections were completed by self-report. For each subject, a score of 0–42 was assigned as the baseline for this truth of depression and anxiety. Higher values ​​indicate greater psychological distress. Scores above 21 and 15 were considered major depression and anxiety, respectively.

### Assessment of anthropometric indicators

Weight was determined to be within 0.1 kg using a digital scale (Seca, Hamburg, Germany) with light clothing and no shoes. The height was also measured within 0.1 cm using a studio meter (Seca) in a steady state without shoes. Body mass index (BMI) was calculated by dividing weight (kg) by height (m2).

### Physical activity assessment

Participants’ physical activity assessment was based on the International Physical Activity Questionnaire (IPAQ) [46]. The IPAQ questionnaire was designed for people aged 15 to 69 years by the WHO and CDC in Geneva in 1998 and is an effective and tested method of physical activity assessment. There are two types of short and long IPAQ questionnaires. In this study, the short form (IPAQ-S) was used. The IPAQ-s consists of 7 questions that assess the frequency and duration of vigorous, moderate, and walking activity, and time spent sitting during the past week. The total score can be expressed as metabolic equivalents (METs). The metabolic rate or oxygen consumed while sitting for one minute is equivalent to one MET (3.5 MI/kg/minute) [47]. This questionnaire was validated (Cronbach’s alpha = 0.7 and test-retest reliability coefficient = 0.9) by Moghaddam et al. in 2012 in Iran [48]. According to the IPAQ guidelines, to calculate the total MET, the minutes spent per week in each category are multiplied by a factor of 8, 4, and 3.3 for vigorous, moderate, and walking activities, respectively [49]. The IPAQ-S was completed through interviews with participants.

### Statistical analysis

Statistical analysis was performed using a computer with SPSS software version 24. Using the Dietitian IV software, we analyzed the results of a 24-hour, 3-day diet call questionnaire to calculate the amount of major and micronutrients. HEI-2015 scores have been converted to quartiles. The distribution of quantitative and qualitative variables across quartiles was evaluated using one-way ANOVA and the chi-square test, respectively. Finally, a step-by-step multivariate linear regression test was used to predict the true relationship between the independent variable and the main study variable. The P value significance level is 0.05.

## Results

**Fig. 1:**
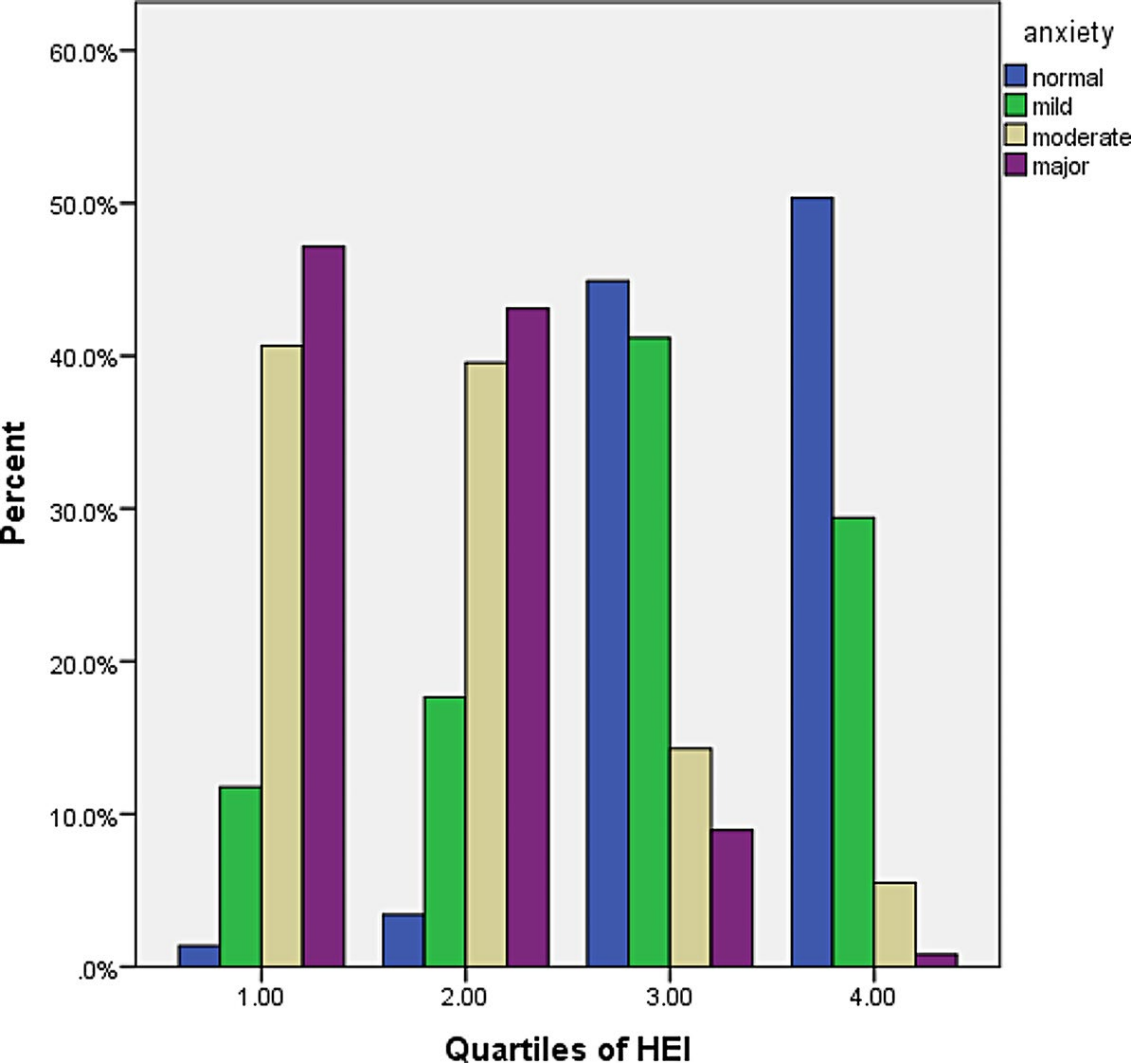
Prevalence of anxiety in study participants across quartiles of the HEI-2015

**Fig. 2:**
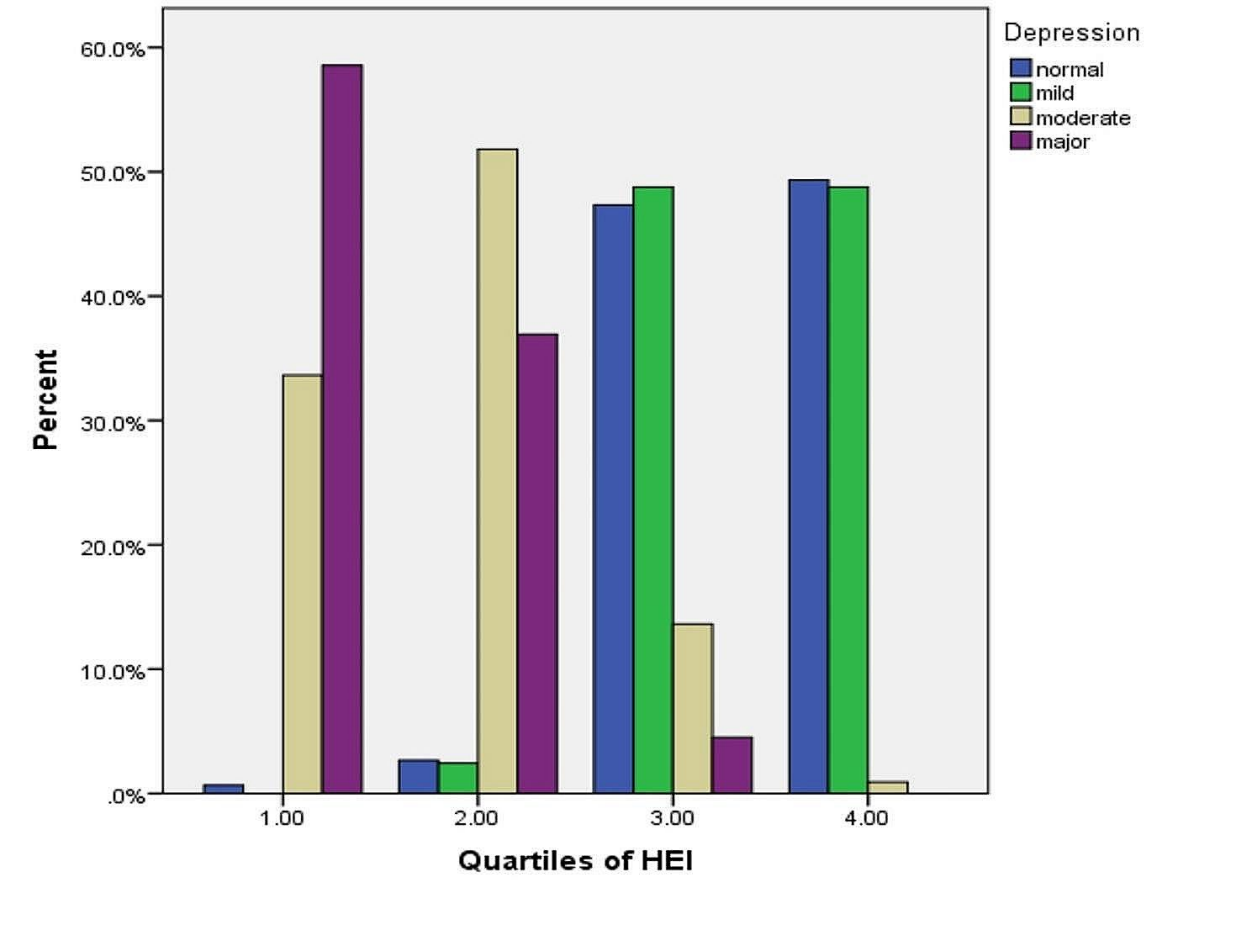
Prevalence of depression in study participants across quartiles of the HEI-2015

The response rate to the questionnaire was 100%. A total of 412 students (mean age 14.83 ± 1.61 years) completed the study. Table 1 shows the baseline characteristics of HEI-2015 quartile participants. Participants with high HEI-2015 scores had a better financial status than participants with low scores. Table 2 shows the food groups for the entire HEI-2015 quartile and the dietary intake of micronutrients. Subjects with high HEI-2015 scores reported higher intakes of whole grains, vegetables, fruits, nuts, and polyunsaturated fatty acids (PUFAs) compared to subjects with lower scores. The prevalence of depression and anxiety in participants was measured at 53.6 and 51.9, respectively. The prevalence of depression and anxiety was lower in subjects with higher HEI-2015 scores (Figs. 1 and 2 ).

**Table 1:** General characteristics of study participants across quartiles (Q) of HEI-2015 (mean values and SD)

		Quartiles of HEI-2015				
		Q1	Q2	Q3	Q4	P value†
n		103	103	111	95	
Age (years)		14.57 ± 1.6	15.05 ± 1.4	15.23 ± 1.5	14.42 ± 1.7	0.54
BMI(kg/m^2^)		22.78 ± 4.7	23.30 ± 5.5	23.73 ± 4.6	21.59 ± 4.1	0.01
Economic status (%)	Low	40	18.9	26.7	14.4	
	Middle	22.7	25.8	23.6	27.9	0.01
	High	16.1	29	35.5	19.4	
Physical activity (%)	Low	44.8	31	13.8	10.3	
	Moderate	27.5	27.2	25.9	19.4	0.01
	High	3.2	11.1	38.1	47.6	
Family size (≤ 4) (%)		26	21.9	20.5	31.5	0.21
Breakfast (%)	Yes	8.0%	17.7%	35.0%	39.2%	0.01
	No	48.0%	34.9%	16.0%	1.1%	
Dietary supplement use (%)	Yes	3.9%	10.0%	41.7%	44.4%	0.01
	No	41.4%	36.6%	15.5%	6.5%	

*p < 0.05 was considered statistically significant

†Obtained from analysis of variance for continuous variables and χ2 test for categorical variables

BMI: body mass index

**Table 2:** Multivariable-adjusted intakes of the selected nutrients in study participants across quartiles (Q) of HEI-2015 (HEI-2015)† (mean values and SD)

Quartiles of HEI-2015									
	Q1		Q2		Q*3*		Q4		
	*n* = 103		*n* = 103		*n* = 111		*n* = 95		
	Mean	SD	Mean	SD	Mean	SD	Mean	SD	p value‡
Energy (kcal/d)	2728.67	521.66537	2922.2293	629.53250	2554.3606	557.81685	2354.3019	357.02692	0.01*
Carbohydrates (g/d)	362.4192	77.43745	379.5286	81.09158	331.1935	77.74061	308.6401	53.32123	0.01*
Proteins (g/d)	81.0844	20.19463	87.7695	21.94103	100.5681	21.03891	98.5135	16.70317	0.01*
Fats (g/d)	115.9769	26.93896	127.3422	35.90509	100.8676	33.56817	89.1825	23.17852	0.01*
Fiber(g/d)	21.09	6.61	24.51	9.75	29.28	8.17	29.71	6.99	0.01*
Trans fat (% of energy)	3	0.83	2.89	0.61	1.13	0.58	1.17	0.46	0.01*
PUFA (% of energy)	27.5691	10.40009	31.9034	12.13324	40.6666	9.98778	41.5151	8.12335	0.01*
Red meat (g/d)	72.77	13.72	84.55	15.03	84.97	15.08	94.26	18.52	0.01*
Whole grains (g/d)	22.32	3.86	27.04	4.69	27.42	6.35	30.44	5.92	0.01*
Fruit (g/d)	274.34	79.71	278.49	36.33	319.86	64.34	321.94	53.17	0.01*
Vegetables (g/d)	233.41	97.12	277.23	11.09	358.52	12.85	359.29	97.03	0.01*
Nuts and legumes (g/d)	16.6862	6.77314	20.4836	9.62591	29.7965	7.44089	30.2491	6.57151	0.01*

*p < 0.05 was considered statistically significant

†Energy intake is adjusted for age and sex; all other values are adjusted for age, sex, and energy intake

‡Obtained from analysis of covariance

After investigating the relationship between HEI and students’ depression and anxiety status, it was observed that some other independent quantitative and qualitative variables, at the same time, have a relatively significant relationship with students’ depression and anxiety status. Now, given the simultaneous effect of these variables on a multivariate logistic regression relationship, first, the question is: Does the relationship between HEI and students’ depression and anxiety remain significant? Second, which variables acted as confounders, and were they removed from the model?

After adjusting for confounders such as the duration of exclusive breastfeeding, simple carbohydrate intake, regular breakfast consumption, and menstrual status, symptoms of depression and anxiety had a significantly negative correlation with the HEI-2015. Tables 3 and 4, show the final result of the multivariate linear regression model.

**Table 3:** Relationship between different variables and depression in linear regression analysis

	R^2^	Standardized Coefficients Beta	Unstandardized Coefficients Beta	P value
HEI	0.795	− 0.532	− 0.014	< 0/0001
Dietary supplement use	0.821	− 0.208	− 0.209	< 0/0001
Dietary Fat	0.828	0.088	0.001	< 0/0001
Physical activity	0.829	− 0.201	− 0.057	< 0/0001

**Table 4:** Relationship between different variables and anxiety in linear regression analysis

	R^2^	Standardized Coefficients Beta	Unstandardized Coefficients Beta	P value
HEI	0.581	− 0.547	− 0.014	< 0/0001
Dietary supplement use	0.602	− 0.183	− 0.184	< 0/0001
Dietary Fat	0.608	0.139	0.002	< 0/0001
Dietary Protein	0.615	− 0.104	− 0.002	< 0/0001

As seen in Table 3, by constantly considering the variables of supplementation, total dietary fat, and physical activity, for a 1 standard deviation (SD) increase in the HEI variable, there is a decrease of 0.5 SD in the depression variable. With constant consideration of HEI variables, total dietary fat, and physical activity, for a 1 SD increase in the supplement consumption variable, we have a decrease of 0.2 SD in the depression variable. By constantly considering the variables of HEI, supplement consumption, and physical activity, per 1 SD increase in total dietary fat, we increase the standard deviation in the depression variable by 0.08. With constant consideration of HEI variables, total dietary fat, and supplement consumption, for 1 standard deviation (SD) in physical activity, there is a decrease of 0.2 SD in the depression variable. In Table 3, the P values are less than 0.05 based on the ANOVA regression table. Thus, the model used is a good predictor of the depression variable. Based on the beta standardized coefficient, all variables except fat showed an inverse relationship with depression. The relationship of the HEI with depression was greater than that of the other variables. HEI variables are approximately 2.5 times more associated with depression than supplementation and physical activity.

As seen in Table 4, by constantly considering the variables of supplementation, total dietary fat, and dietary protein, for a 1 standard deviation (SD) increase in the HEI variable, there is a decrease of 0.5 SD in the anxiety variable. With constant consideration of HEI variables, total dietary fat, and dietary protein, for a 1 SD increase in the supplement consumption variable, we have a decrease of 0.1 SD in the anxiety variable. By constantly considering the variables of HEI, supplement consumption, and dietary protein, per 1 SD increase in total dietary fat, we increase the standard deviation in the anxiety variable by 0.13. With constant consideration of HEI variables, total dietary fat, and supplement consumption, for 1 standard deviation (SD) in dietary protein, there is a decrease of 0.1 SD in the anxiety variable. In Table 4, the P values are less than 0.05 based on the ANOVA regression table. Thus, the model used is a good predictor of the anxiety variable. Based on the beta standardized coefficient, all variables except dietary fat showed an inverse relationship with anxiety. The relationship of HEI with anxiety was greater than that of the other variables. HEI variables are approximately 3 times more associated with anxiety than supplementation and 5 times more associated with dietary protein.

## Discussion

This study was performed on female high school students in the city of Babol and aimed to investigate the relationship between HEI and the incidence of depression and anxiety in adolescents. The findings of this study showed that the HEI correlated with depression and anxiety scores (*p* ≤ 0.05).

The results of this study showed that all four variables of HEI, dietary supplements, dietary fat, and physical activity are highly correlated with depression. HEI, dietary supplements, dietary fat, and protein were related to anxiety scores (*p* ≤ 0.05), and these factors can explain the changes in depression, strongly, but on medium, in anxiety. Based on the current study, HEI, dietary supplements, and physical activity seem to have a strong inverse correlation with depression, and with increasing values of these three variables, the rate of depression decreases significantly. The same is true of anxiety. Based on the current study, HEI, dietary supplement, and dietary protein seem to have a strong inverse correlation with anxiety, and with increasing values of these three variables, the rate of anxiety decreases significantly.

Nutrition is one of the influential factors in depression and anxiety [50]. HEI is an important indicator for assessing overall dietary diversity. Previous studies have shown that a higher HEI is related to higher nutrient intake and the adequacy of the diet [51]. In the present study, the HEI was found to correlate significantly with depression and anxiety. Our findings support studies that show a link between overall diet and mental health, rather than individual nutrients and foods [51]. An inverse relationship has been demonstrated between depression and a healthy dietary pattern with a diet rich in vegetables, fruits, nuts, legumes, olive oil, fish, whole grains, and moderate consumption of red and processed meat [52–54]. Based on a cohort study by Godos et al. polyphenol classes, specifically flavonoids and phenolic acids, are conversely associated with depressive symptoms [55], and it has been shown that diets high in plant foods, including fruits and vegetables, which contain the highest variety of polyphenols [56], and legumes, plant proteins, and whole grains, are rich in flavonoids [57]. On the other hand, the HEI-2015 diet pattern also measures fruits, vegetables, legumes, plant proteins, and whole grain consumption in the diet [58]. Therefore, it can be said that the HEI dietary pattern measures a high intake of polyphenol-rich foods.

In addition, Western dietary patterns (characterized by processed or fried foods, refined grains, and sugar-rich products) are at increased risk of depression and anxiety [59]. These diets lead to poor spatial learning and memory performance associated with hippocampal function. In addition, studies have shown that a Western diet reduces brain-derived neurotrophic factor (BDNF), which is not associated with obesity or malnutrition, in the short term [60]. BDNF protects neurons from oxidative stress and facilitates neurogenesis [61]. Given that the neurological and cognitive effects of BDNF are greater in adolescence than in adulthood, a healthy diet at this stage of life is more beneficial to hippocampal growth and function than at later stages. There is a possibility. Therefore, dietary changes made during adolescence can have longer-lasting effects than those that begin in adulthood [28]. Therefore, diet can affect a person’s state of mind by changing and adjusting the proportion of BDNF.

On the other hand, a high-fat diet (HFD) causes obesity-related metabolic disorders characterized by mild inflammation. Chronic inflammation impairs cell function, alters metabolism, and causes pathological conditions [62]. Evidence suggests that HFD-dependent neuroinflammation adversely affects BDNF signaling and mitochondrial activity in the cerebral cortex [62]. HFD in mice also has detrimental effects on the bioenergetics of the brain cortex. HFD alters mitochondrial function, performance, and oxidative stress. According to some evidence, presynaptic mitochondrial dysfunction may be one of the main mechanisms leading to cognitive impairment in neurological disorders [63]. Previous studies of civilians have shown a direct link between adherence to the Western diet and risk of mental illness [64]. Increased intake of fruits, vegetables, and whole grains has been reported to be associated with a reduced risk of anxiety. Conversely, a diet high in refined grains and sweet or fried foods was associated with increased anxiety levels [59], as reported in our study.

Research has also found that diet plays an important role in the composition of the gut microbiota [65], and dietary changes help reverse the negative effects of stress on the microbiome [28]. Although the effect of diet on the microbiome in adolescence has not yet been studied, it can be said that lifestyle and diet changes in adolescence have an effect on the intestinal microbiome and therefore can be related to the regulation of neurogenesis in the hippocampus and related cognitive function in this period [62].

In addition, potential mechanisms were recommended to clarify the reverse association of HEI-2015 with depression. High concentrations of antioxidants, such as folic acid and vitamin E in HEI-2015, may protect neurons from the degenerative effects of inflammation and oxidative stress [66]. HEI-2015 is also high in omega-3s and PUFAs with potential neuroprotective effects [67]. The Iranian diet is also rich in refined grains, rice, and hydrogenated oil [68]. In addition, the high score of HEI-2015 indicates high planet food consumption, which is rich in flavonoids and fiber [69]. The consumption of plant-based foods rich in fiber and polyphenols alters the gut microbiome by creating microbial diversity in the gut that has an association with a decreased risk of chronic diseases [69], so a possible mechanism may be to alter the gut microbiota by eating fiber-rich foods.

Dietary supplement use, in line with this study, other research conducted in Australia, Italy, and the United States have reported a complementary role in preventing mental diseases and controlling symptoms, such as aggression, insomnia, hallucinations, and depression. This association is due to the important role of these compounds as constituent coenzymes of neuronal precursors (gamma-amino-butyric acid, serotonin, etc.) [70, 71].

Extensive research has investigated the association between physical activity and depression and anxiety. This study found a significant relationship between physical activity and depression and anxiety. Bhui concluded that people participating in at least 92 min a day of exercise compared to those participating in 0–44 min a day of physical activity experienced less anxiety because physical activity can play an effective role in reducing mental disorders by reducing inflammation and changing neurochemical pathways [72, 73]. Another possible mechanism is the effect of physical activity on the amount of amine neurotransmitters. Studies have shown that people with mental illness have lower levels of serotonin, dopamine, and norepinephrine and that physical activity increases the levels of neurotransmitters [74].

Potential limitations of this study included a cross-sectional design that made it difficult to identify causal relationships. The 24-hour nutrition questionnaire was a major limitation of our study and had several limitations, including difficulty remembering food consumed, exact portion size, and underreporting or overreporting. Despite these limitations, this study was the first to examine the association between HEI and depression and anxiety in adolescents. Inclusion and exclusion criteria were clearly defined, and many potential confounding factors were tested. Adolescent studies examining the relationship between nutrition and mental health, in general, have shown that the relationship is complex and potentially bidirectional [75]. Given brain development in childhood and adolescence and the onset of depression in adolescence, the effect of diet during this period on mental health is likely to be greater than in old age [76]. On the other hand, a closer look at the diets of depressed people reveals an interesting observation. Their diet is not adequate. They choose the wrong foods and choose foods that can contribute to depression [77].

Furthermore, several other factors, such as socioeconomic status (SES), family income, and education level, also influence food choices, and depression may also lead to reduced food intake [78].

## Conclusion

Our results revealed a significant inverse relationship between HEI and depression and anxiety in adolescents. Our results demonstrate the importance of maintaining a more varied diet and important public health implications for the prevention of depression and anxiety. Further epidemiological studies are recommended to fully identify such associations, along with prospective cohort studies to potentially determine causality.

## Data Availability

The datasets used and analyzed during the current study are available from the corresponding author on reasonable requests.

## Abbreviations

BMI: Body mass index
HEI: Healthy eating index
LMIC: Low-to Middle-Income Country
HIC: High-Income Country
IPAQ: International Physical Activity Questionnaire
SES: Socioeconomic status
HFD: High-fat diet
BDNF: Brain-derived neurotrophic factor
PUFAs: Polyunsaturated fatty acids
DASS: Depression, Anxiety, and Stress Scales
